# Postoperative radiotherapy is associated with improved survival in pT1-2N1 oral and oropharyngeal cancer without adequate neck dissection

**DOI:** 10.1186/s13014-020-01736-8

**Published:** 2021-01-06

**Authors:** Ching-Chieh Yang, Bor-Hwang Kang, Wen-Shan Liu, Chun-Hao Yin, Ching-Chih Lee

**Affiliations:** 1 Department of Radiation Oncology, Chi-Mei Medical Center, Tainan, Taiwan; 2 Department of Pharmacy, Chia-Nan University of Pharmacy and Science, Tainan, Taiwan; 3 Department of Otolaryngology, Head and Neck Surgery, Kaohsiung Veterans General Hospital, Kaohsiung, Taiwan; 4 School of Medicine, National Defense Medical Center, Taipei, Taiwan; 5 Department of Pharmacy, Tajen University, Pingtung, Taiwan; 6 Department of Radiaton Oncology, Kaohsiung Veterans General Hospital, Kaohsiung, Taiwan; 7 Department of Medical Education and Research, Kaohsiung Veterans General Hospital, Kaohsiung, Taiwan; 8 Institute of Hospital and Health Care Administration, National Yang-Ming University, Taipei, Taiwan

**Keywords:** Oral cancer, Oropharyngeal cancer, Radiotherapy, Neck dissection, Survival

## Abstract

**Background:**

To assess the benefit of postoperative radiotherapy in patients with pT1-2N1M0 oral and oropharyngeal cancer by the quality of neck dissection.

**Methods:**

In the Surveillance, Epidemiology, and End Results database, pT1-2N1M0 oral and oropharyngeal cancer patients treated by primary tumor resection and neck dissection with or without radiotherapy were included between 2004 and 2015. Univariate and multivariate analysis were used to explore the effect of adjuvant radiotherapy on 5-year overall survival (OS) and disease-specific survival (DSS) among different quality of neck dissection.

**Results:**

Of the 1765 patients identified, 1108 (62.8%) had oral cancer, 1141 (64.6%) were men, and 1067 (60.5%) underwent adjuvant radiotherapy. After adjusting for confounding factors, postoperative radiotherapy reduced the adjusted hazard ratio (aHR) of 5-year OS to 0.64 (95% confidence interval [CI] 0.49–0.84) in those with < 18 lymph nodes (LNs) removed, but not in those with 19–24 LNs removed (aHR 0.78; 95% CI 0.73–1.13), and in those with ≥ 25 LNs removed (aHR 0.96; 95% CI 0.75–1.24). For 5-year DSS, similar effect was observed. The adjusted hazard ratio was 0.66 (95% confidence interval, 0.45–0.97) in those with < 18 LNs. The protective effect was not seen in those with 18–24 LNs (aHR 1.07; 95% CI 0.59–1.96), and in those with ≥ 25 LNs (aHR 1.12; 95% CI 0.81–1.56). Sensitivity testing also showed a robust protective effect of postoperative radiotherapy in patients with < 18 LNs removed.

**Conclusion:**

Radiotherapy was associated with improved survival in pT1-2N1M0 oral and oropharyngeal cancer patients without adequate neck dissection.

## Background

Oral and oropharyngeal cancer remain among the most common malignancies in the world [1]. Although most early stage oral and oropharyngeal patients have a favorable prognostic outcome, previous literatures have reported locoregional recurrence in 30–35% of patients, and around 20% will eventually die of the disease [2, 3]. The use of postoperative radiotherapy could improve tumor control and survival in those with advanced local disease (T3–4), close or positive margins, perineural or vascular invasion, multiple positive lymph nodes (LNs) (N2–3), and extracapsular extension [4]. However, the routine use of postoperative radiotherapy for T1–2 stage disease limited to a single, ipsilateral positive LN not larger than 3 cm (i.e., N1) without adverse features remains controversial [5].

Cervical positive LNs, those most commonly related to recurrence, are recognized as among the most important prognostic factors in head and neck cancer [6]. Recent studies have confirmed that the number of evaluated LNs correlates with outcomes [7, 8]. Ebrahimi et al. reported that an LN yield < 18 was associated with worse 5-year overall (hazard ratio [HR], 2.0; 95% confidence interval [CI] 1.1–3.6), disease-specific (HR 2.2; 95% CI 1.1–4.5), and disease-free survival (HR 1.7; 95% CI 1.1–2.8). Thus, LN yield ≥ 18 has been proposed as a cut-off point for good-quality neck dissection. Inadequate LN harvests may lead to stage migration and subsequent underestimation of disease severity [9, 10]. Although postoperative radiotherapy was considered in those with pN1 disease, scant studies investigated the survival benefit of postoperative radiotherapy for those without adequate LN dissection.

Thus, in this study, we used data from the Surveillance, Epidemiology, and End Results (SEER) database to assess the relationship between postoperative radiotherapy and outcomes by quality of neck dissection in oral and oropharyngeal cancer patients with pT1-2N1 disease.

## Methods

### Data source and study population

**Fig. 1:**
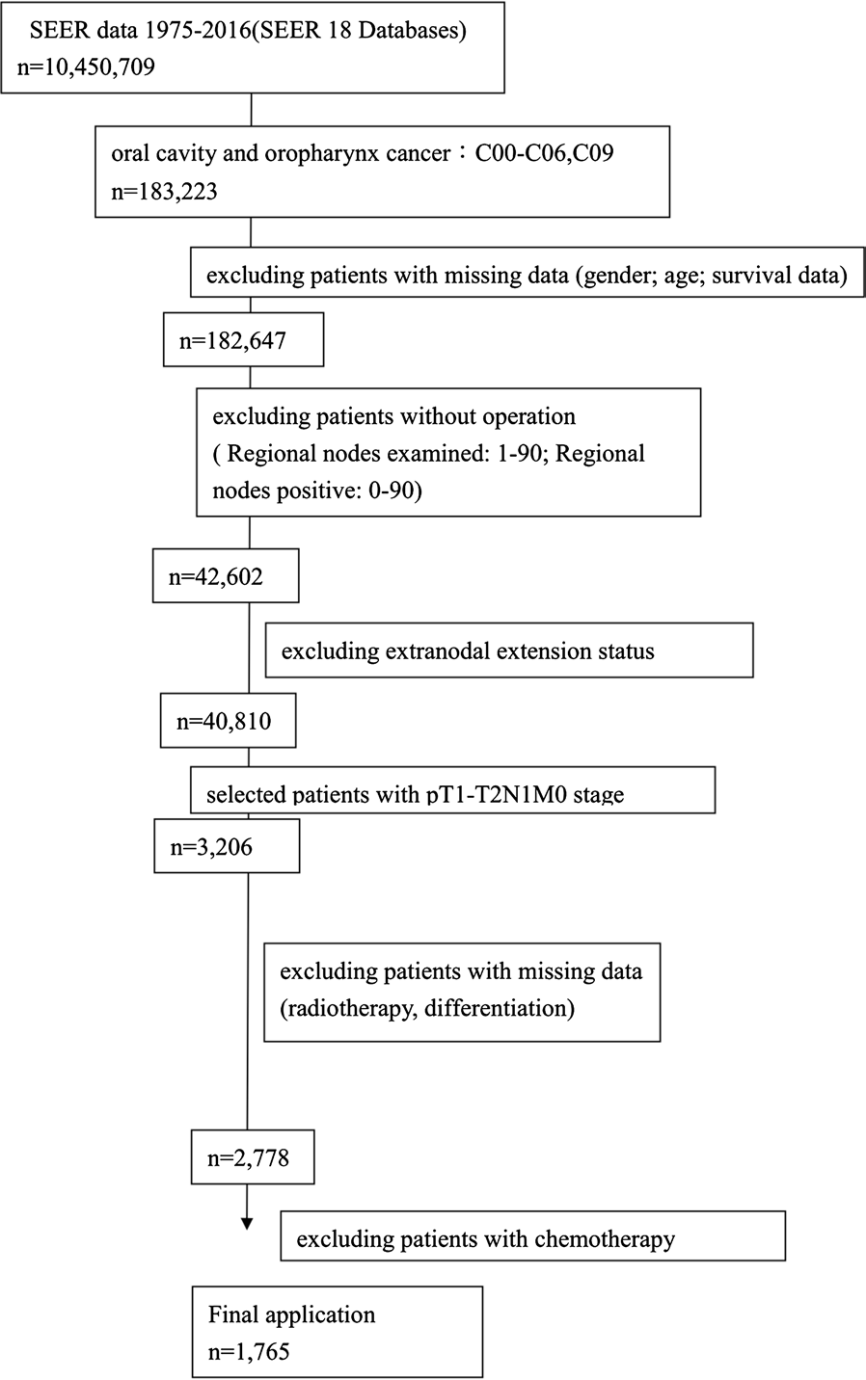
Patient selection flowchart

SEER is an open access resource of data on patients from the United States for cancer-based epidemiology analyses. Data identification and extraction is done using the Surveillance Research Program, National Cancer Institute SEER*Stat software (seer.cancer.gov/SEER*stat) Version 8.3.2. Patients with newly-diagnosed oral and oropharyngeal cancer post primary tumor resection and neck dissection with pathological pT1-2N1M0 were identified from 2004 to 2015. Patients were identified using the International Classification of Disease for Oncology, third edition (ICD-O-3) codes for oral cavity (C01–C06; C14) and oropharynx (C09–C10). These patients were staged according to the 6th or 7th edition American Joint Committee on Cancer (AJCC) classification system corresponded to the year of diagnosis [11]. Between the 6th or 7th edition AJCC staging, there were no changes made to the classification criteria of T1–2 oral and oropharyngeal SCC or the N classification criteria. Due to there is no information about extranodal extension or p16 status in the SEER database, 8th edition AJCC staging was not adapted in current analysis. Patients with missing data such as age, gender, radiotherapy, clear AJCC TNM stage, and follow-up data were excluded. Finally, a total of 1765 patients were recruited into this analysis (Fig. 1). Patients were divided into those who received radiotherapy within six months of surgery (the postoperative radiotherapy group) and those who did not (the observation group). The end points were the 5-year overall survival (OS) and disease-specific survival (DSS) rates. Deaths due to cancer were recorded as events and deaths secondary to other causes, at 5 years following diagnosis or the last follow up date, were recorded as censored.

### Statistical analysis

All statistical operations were performed using SPSS (version 15, SPSS Inc., Chicago, IL, USA). Categorical variables were compared with Pearson’s chi-square test or Fisher’s exact test. Differences in continuous variables were analyzed with one-way ANOVA. The Kaplan–Meier method was used to estimate the 5-year OS and DSS rates for the postoperative radiotherapy and the observation group. In multivariate Cox regression analyses, the therapeutic effect of postoperative radiotherapy was analyzed after adjusting for all variables (age, gender, pathological T classification, tumor differentiation, year of diagnosis, marital status and insurance). Because the target area of postoperative radiotherapy could not be extracted from the SEER database, we assumed that some oral and orophargneal cancer patients might receive postoperative radiotherapy due to pathological risk features of the primary tumor, instead of nodal disease. We hope to exclude those who underwent postoperative radiotherapy only on the primary tumor site. The positive margin has been reported to be around 1.6% in early stage oral cancer surgery [12]. In T1-2 oral cancer patients, the rate of lymphovascluar permeation or perineural invasion is 13.3% [12]. Thus, a sensitivity test was used to evaluate the association between postoperative radiotherapy and survival outcomes [13]. We randomly excluded 15% patients who underwent postoperative radiotherapy which might due to adverse pathological features. The remaining 85% patients with postoperative radiotherapy were merged with those without postoperative radiotherapy for further analysis. A two-sided *P *value (*P* < 0.05) was considered significant.

### Results

As shown in Table 1, a total of 1765 newly-diagnosed oral and oropharyngeal cancer patients treated with primary tumor resection and neck dissection were included, 1108 (62.8%) diagnosed with oral cancer and 657 (37.2%) diagnosed with oropharyngeal cancer. Of these, 1141 (64.6%) were men, the mean (standard deviation) age was 60.9 (12.4) years, and 1067 (60.5%) underwent adjuvant radiotherapy. Patients with < 18 LNs retrieved were more likely to be older and have pT1 disease.

**Table 1:** Demographic and clinical characteristics of the oral and oropharyngeal cancer patients, *n* = 1765

Variable	Total	18 < LNs	18 ≦ LNs < 25	25 ≦ LNs	*P* value
	*n* = 1765	*n* = 598	*n* = 289	*n* = 878	
RT	1067 (60.5)	354 (59.2)	184 (63.7)	529 (60.3)	0.436
**Gender**					
Female	624 (35.4)	230 (38.5)	100 (34.6)	294 (33.5)	0.139
Male	1141 (64.6)	368 (61.5)	189 (65.4)	584 (66.5)	
Age, years (Mean ± SD)	60.9 ± 12.4	62.8 ± 12.6	60.7 ± 11.9	59.7 ± 12.2	< 0.001
**Pathological T classification**					
T1	883 (50.0)	332 (55.5)	138 (47.8)	413 (47)	0.004
T2	882 (50.0)	266 (44.5)	151 (52.2)	465 (53)	
**Differentiation**					
Well	166 (9.4)	65 (10.9)	21 (7.3)	80 (9.1)	0.095
Moderately	1021 (57.8)	329 (55)	186 (64.4)	506 (57.6)	
Poorly	578 (32.7)	204 (34.1)	82 (28.4)	292 (33.3)	
**Site**					
Oral cavity	1108 (62.8)	360 (60.2)	185 (64)	563 (64.1)	0.277
Oropharynx	657 (37.2)	238 (39.8)	104 (36)	315 (35.9)	

*LNs* lymph nodes, *RT* radiotherapy

**Fig. 2:**
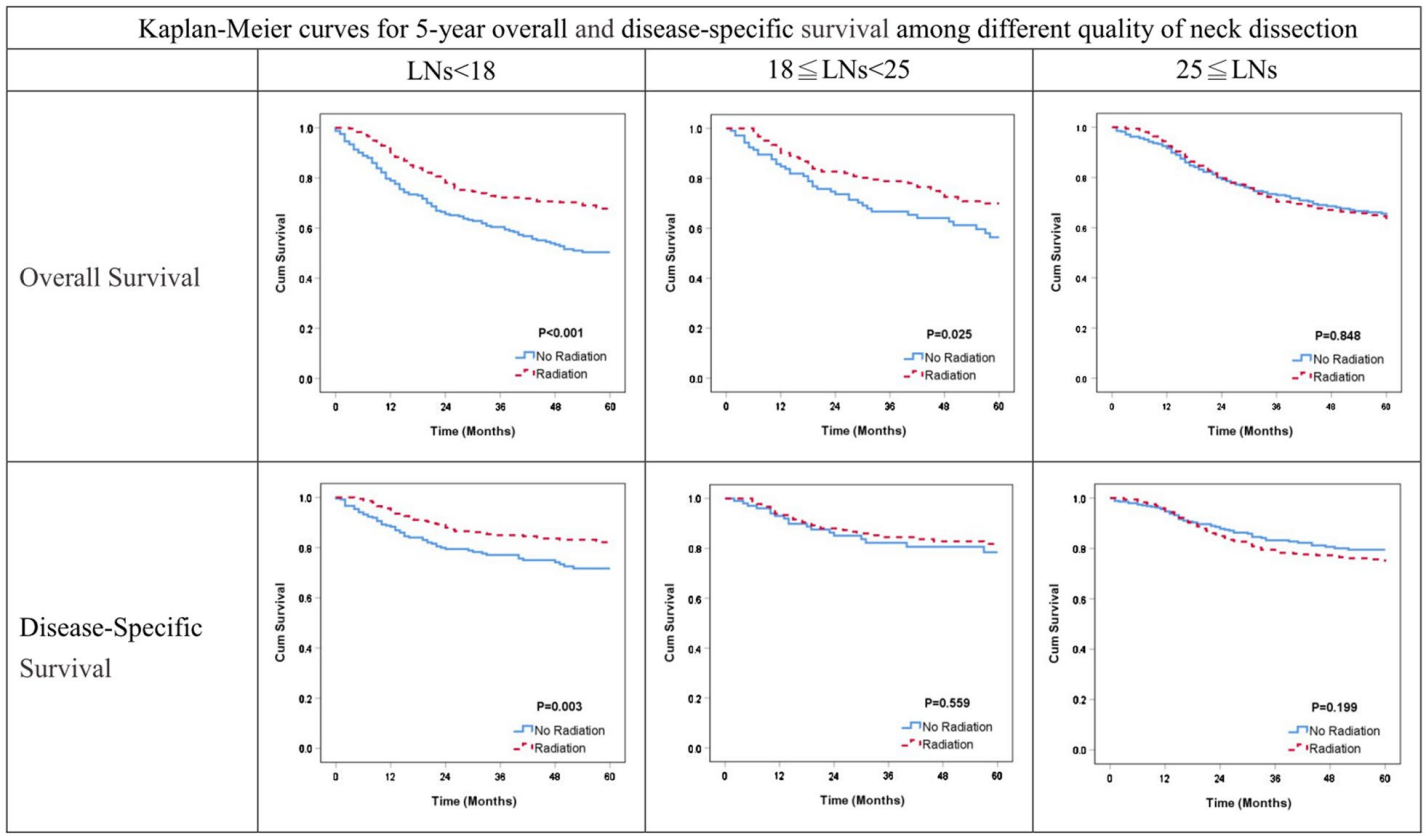
Kaplan–Meier curves for 5-year overall and disease-specific survival among different quality of neck dissection

Table 2 and Fig. 2 illustrate the impact of postoperative radiotherapy on 5-year OS and DSS according to the quality of the retrieved LNs (poor-quality: < 18 LNs retrieved; good-quality: 18–24 LNs retrieved; high-quality: ≥ 25 LNs retrieved). Compared to the observation group, the postoperative radiotherapy group had better 5-year OS and DSS rates (all, *P* < 0.05) in those with poor-quality neck dissection (< 18 LNs retrieved) and had better 5-year OS rate (*P* = 0.025) in patients with good-quality neck dissection (18–24 LNs retrieved). After adjusting for all confounding factors, the impact of postoperative radiotherapy was as follows: for those with < 18 LNs retrieved, the 5-year OS adjusted hazard ratio (aHR) was 0.64 (95% confidence interval [CI], 0.49–0.84); for those with 18–24 LNs retrieved, the aHR was 0.78 (95% CI 0.73–1.13); for those with ≥ 25 LNs retrieved, the aHR was 0.96 (95% CI 0.75–1.24). For 5-year DSS, in those with < 18 LNs retrieved, the aHR was 0.66 (95% CI 0.45–0.97); for those with 18–24 LNs retrieved, the aHR was 1.07 (95%, 0.59–1.96); for those with > 25 LNs retrieved, the aHR was 1.12 (95% CI 0.81–1.56) (Table 3). Postoperative radiotherapy had a protective effect on survival in patients with poor-quality neck dissection (< 18 LNs retrieved). Stratified analyses separating oral and oropharynx cancer patients were also performed (Supplementary Table1, 2 and 3). Sensitivity test showed that the protective effect of postoperative adjuvant radiotherapy remained robust in those with poor-quality neck dissection (< 18 LNs retrieved) alone (aHR 0.61; 95% CI 0.45–0.81) (Table 4).

**Table 2:** The relationship between postoperative radiotherapy and observation groups in 5-year overall and disease-free survivals among different quality of neck dissection, *n* = 1765

	LNs < 18				18 ≦ LNs < 25				25 ≦ LNs			
	Total	Event (%)	Survival rate (%)	*P* value	Total	Event (%)	Survival rate (%)	*P* value	Total	Event (%)	Survival rate (%)	*P* value
**Overall survival**	598	215 (36.0)	60.7	< 0.001	289	88 (30.5)	65.1	0.025	878	263 (30.0)	64.1	0.848
Without RT	244	111 (45.5)	50.4		105	40 (38.1)	56.4		349	103 (29.5)	65.1	
With RT	354	104 (29.4)	67.8		184	48 (26.1)	70.0		529	160 (30.3)	63.4	
**Disease-specific survival**	598	109 (18.2)	78.0	0.003	289	47 (16.3)	80.6	0.559	878	164 (16.7)	76.7	0.199
Without RT	244	56 (23.0)	71.7		105	18 (17.1)	78.5		349	57 (16.3)	79.5	
With RT	354	53 (15.0)	82.1		184	29 (15.8)	81.8		529	107 (20.2)	74.8	

*LNs* lymph nodes, *RT* radiotherapy

**Table 3:** Multivariate analyses of risk factors regarding 5-year overall and disease-free survivals using Cox regression model among different quality of neck dissection, *n* = 1765

	LNs < 18		18 ≦ LNs < 25		25 ≦ LNs	
	HR (95% CI)	*P* value	HR (95% CI)	*P* value	HR (95% CI)	*P* value
**Overall survival**						
With RT	0.64 (0.49–0.84)	0.001	0.73 (0.48–1.13)	0.158	0.96 (0.75–1.24)	0.769
Male	0.86 (0.66–1.13)	0.279	1.01 (0.64–1.60)	0.961	1.08 (0.83–1.40)	0.557
Age	1.03 (1.02–1.05)	< 0.001	1.04 (1.02–1.06)	< 0.001	1.01 (1.002–1.02)	0.022
Pathological T classification: T2	1.62 (1.23–2.12)	0.001	1.89 (1.21–2.96)	0.005	1.72 (1.34–2.22)	< 0.001
Differentiation: poorly	1.11 (0.81–1.52)	0.515	1.29 (0.80–2.10)	0.301	1.18 (0.89–1.55)	0.246
Site: oropharynx	0.43 (0.30–0.62)	< 0.001	0.46 (0.26–0.82)	0.008	0.34 (0.24–0.47)	< 0.001
**Disease-specific survival**						
With RT	0.66 (0.45–0.97)	0.033	1.07 (0.59–1.96)	0.822	1.12 (0.81–1.56)	0.491
Male	0.74 (0.51–1.08)	0.122	1.08 (0.58–2.01)	0.820	1.01 (0.73–1.40)	0.957
Age	1.02 (1.001–1.03)	0.043	1.03 (1.004–1.06)	0.023	1.00 (0.99–1.01)	0.715
Pathological T classification: T2	1.54 (1.06–2.25)	0.025	2.27 (1.21–4.26)	0.011	1.54 (1.12–2.12)	0.008
Differentiation: poorly	1.26 (0.82–1.93)	0.297	1.68 (0.88–3.22)	0.117	1.34 (0.95–1.89)	0.098
Site: oropharynx	0.33 (0.19–0.55)	< 0.001	0.19 (0.07–0.50)	0.001	0.28 (0.18–0.44)	< 0.001

*LNs* lymph nodes, *RT* radiotherapy

*Adjusted for age, gender, pathological T classification, differentiation, site, race, marital and insurance

**Table 4:** Multivariate analyses of risk factors regarding 5-year overall and disease-free survivals using Cox regression model among different quality of neck dissection, a sensitivity test (85%), *n* = 1605

	LNs < 18		18 ≦ LNs < 25		25 ≦ LNs	
	HR (95% CI)	*P* value	HR (95% CI)	*P* value	HR (95% CI)	*P* value
**Overall survival**						
With RT	0.61 (0.45–0.81)	0.001	0.70 (0.45–1.11)	0.128	0.99 (0.76–1.28)	0.931
Male	0.88 (0.66–1.18)	0.397	1.14 (0.69–1.87)	0.606	1.06 (0.81–1.39)	0.672
Age	1.04 (1.03–1.05)	< 0.001	1.04 (1.02–1.06)	0.001	1.01 (1.001–1.02)	0.038
Pathological T classification: T2	1.70 (1.28–2.25)	< 0.001	1.88 (1.17–3.03)	0.009	1.69 (1.30–2.20)	< 0.001
Differentiation: poorly	1.10 (0.79–1.54)	0.562	1.27 (0.77–2.10)	0.357	1.20 (0.90–1.61)	0.209
Site: oropharynx	0.48 (0.33–0.69)	< 0.001	0.52 (0.29–0.93)	0.028	0.33 (0.23–0.47)	< 0.001
**Disease-specific survival**						
With RT	0.62 (0.41–0.93)	0.020	1.06 (0.57–1.99)	0.848	1.20 (0.86–1.67)	0.297
Male	0.76 (0.51–1.13)	0.169	1.12 (0.58–2.19)	0.732	0.97 (0.70–1.36)	0.868
Age	1.02 (1.01–1.04)	0.011	1.03 (0.99–1.06)	0.054	1.00 (0.98–1.01)	0.696
Pathological T classification: T2	1.65 (1.11–2.46)	0.013	2.01 (1.04–3.91)	0.039	1.49 (1.07–2.08)	0.017
Differentiation: poorly	1.24 (0.79–1.96)	0.353	1.71 (0.87–3.35)	0.119	1.38 (0.97–1.98)	0.077
Site: oropharynx	0.37 (0.21–0.63)	< 0.001	0.17 (0.06–0.48)	0.001	0.29 (0.19–0.46)	< 0.001

*LNs* lymph nodes, *RT* radiotherapy

*Adjusted for age, gender, pathological T classification, differentiation, site, race, marital and insurance

## Discussion

In contrast to the latest NCCN guidelines which suggest adjuvant radiotherapy for patients with pT1-2N1 disease, our results demonstrated that postoperative radiotherapy was effective only in those without good-quality neck dissection (LN yield < 18) [4, 14]. The protective effect of adjuvant radiotherapy failed to reach statistical significance for those with LN yield ≥ 18. Because of the protective role of postoperative radiotherapy in oral and oropharyngeal cancer patients with pT1-2N1M0 disease without good-quality neck dissection, the NCCN guidelines for adjuvant radiotherapy in pN1 disease without adverse features may need to be modified according to the quality of retrieved LNs.

Several studies have demonstrated that pN1 may itself be an indication for postoperative radiotherapy after resection of oral cavity primary tumors [5, 15]. Chen et al. reported an association of postoperative radiotherapy with improved OS for 1467 patients from the National Cancer Database with oral (HR 0.76; 95% CI 0.63–0.92) and oropharyngeal cancer (HR 0.62; 95% CI 0.41–0.92) with pN1 disease without adverse feature, especially in those younger than 70 years or those with pT2 disease. Shrime et al. [16], in an analysis of 1539 patients with T-21N1 oral cancer, found that 78.6% had postoperative radiotherapy, which was associated with better 5-year OS (54.2% vs 41.4%, *P* < 0.001). However, from the same SEER database, Kao et al. [17] found no significant difference in 5-year OS between patients with N1 oral cancer with and without postoperative radiotherapy (38.7% vs 36.0%, *P* = 0.23). One explanation for these different results may be that inadequate LN harvests could lead to stage migration and subsequent underestimation of disease severity, especially in those with pN1disease [6]. To our knowledge, our study is the first to evaluate the benefit of postoperative radiotherapy by quality of neck dissection in patients with pN1 involvement and no risk factors. Moreover, our large cohort (n = 1765) indicates that the effect of operative radiotherapy on survival could assist clinicians in their therapeutic planning for these patients.

Because cervical LN metastasis significantly worsens the prognosis of patients with primary head and neck cancer by 50%, in general, the status of LN metastasis must be known for proper treatment [3]. Many studies have used LN count as a prognostic factor in head and neck cancer patients and also as a potential quality metric for neck dissection [7, 9]. Divi et al. [9] examined these associations in a large cohort from the U.S. National Cancer Database. They found an independent and significant association between examining < 18 LNs and increased risk of death (hazard ratio [HR] 1.18; 95% CI 1.13–1.22). When patients were stratified by clinical nodal stage, the hazard of death was increased in both node negative and node positive groups (HR 1.24; 95% CI 1.17–1.32; HR 1.12; 95% CI 1.05–1.19, respectively). The study found a significant overall survival advantage when > 18 LNs are examined after neck dissection, concluding that 18 LNs is an effective cutoff for improved survival.

Although several studies have outlined the importance of LN yield on head and neck cancer, few have discussed the association of postoperative adjuvant radiotherapy with the quality of neck dissection [18, 19]. According to the latest NCCN guidelines, postoperative adjuvant radiotherapy may be considered in those with pN1 disease without other risk features [4]. Our study explored the impact of postoperative radiotherapy in patients with pT1-2N1M0 without extranodal extension stratified by the number of retrieved LNs. For patients without good-quality neck dissection (LN yield < 18), postoperative adjuvant therapy could reduce the mortality rate 40%. However, patients with good-quality neck dissection (LN yield ≥ 18) did not have a statistically significant reduction in 5-year OS or DSS (Tables 3, 4). Thus, our results recommended that postoperative radiotherapy is encouraged to use for pT1-2N1 patients without adequate neck dissection (LN yield < 18).

At present, treatment protocols for head and neck cancer, such NCCN guidelines, incorporate the TNM stage, surgical margin, pathological adverse features, and response to chemotherapy or radiotherapy [4]. Margin status could be regarded as a proxy of surgery quality for primary tumor resection, although compartment surgery more than wide resection had better outcomes [20]. However, neck dissection quality is not included in the treatment guidelines, even though retrieving more than 10 LNs in elective neck dissection and more than 15 in radical neck dissection has been suggested in order to prevent stage migration [6]. For head and neck patients with pN1 disease, reports of the association between disease outcomes and adjuvant radiotherapy are conflicting [5, 11, 15, 16]. The confusion may stem in part from the heterogenous pattern of pN1 disease (which ranges from microscopic disease to 3 cm LNs), the extent of extracapsular spread, the various cancer subsites, and the quality of the neck dissection. In 2019, an expert panel suggested adjuvant radiotherapy in oral cancer patients with pN1 disease without good-quality neck dissection (< 18 LNs) and recommended the conduct of further prospective clinical trials [21, 22]. Our report provides evidence for the therapeutic effect of adjuvant radiotherapy for patients with pT1-2N1M0 oral cancer. Among patients without good-quality neck dissection, adjuvant radiotherapy could reduce the mortality rate up to 40% (aHR 0.61; 95% CI 0.48–0.78). Radiotherapy appears to offset the negative effect of poor-quality neck dissection and its therapeutic effect decreased as the number of retrieved LNs increased (Fig. 2).

There are several limitations in our series. First, the radiation field was not clearly described in the database. The radiation field might include the primary tumor, regional neck area, or both. These data could be not be extracted from the SEER database. Second, we tried to explore the effect of postoperative radiotherapy in pT1-2N1M0 patients and assumed that the radiotherapy was probably directed to the neck region for pN1 status. However, postoperative radiotherapy could be applied to the primary site due to the pathological features of the primary tumor. There is no information about extanodal extension, lymphovascular permeation, perineural invasion and depth of tumor invasion in the SEER database. The percentage of postoperative radiotherapy that might be considered in those with positive margin, lymphovascular permeation or perineural invasion was 15% [12]. In sensitivity testing in our study, the effect of postoperative radiotherapy remained robust among patients without good-quality neck dissection, when the association of margin status, lymphovascluar permeation, or perineural invasion in the primary tumor were considered. Third, the study included only pT1-2N1M0 patients. Generalization of these results to other oral cancer patients, such as those with T3-4N1M0, will require additional studies. Finally, due to lacking information about extranodal extension or p16 status, 8th edition of AJCC staging system was not adapted in current analysis. Utilizing the latest AJCC staging system is necessary to valid our hypothesis in the future work.

## Conclusions

Oral and oropharyngeal cancer patients with pT1-2N1M0 have a generally favorable prognostic outcome. However, inadequate LN harvest may lead to underestimation of disease severity. Our results suggest that postoperative radiotherapy should be considered for patients without adequate neck dissection. Modification of NCCN guidelines according to the number of retrieved LNs for postoperative radiotherapy in pN1 disease without an adverse feature may be appropriate.

## Supplementary Information

**Additional file 1:** The relationship and multivariate analyses regarding 5-year overall and disease-free survivals between different quality of neck dissection.

## Data Availability

The datasets used during the present study are available from the corresponding author upon reasonable request.

## Abbreviations

NCCN: National Comprehensive Cancer Network
LNs: Lymph nodes
SEER: Surveillance, Epidemiology, and End Results
ICD-O-3: International Classification of Disease for Oncology, third edition
AJCC: American Joint Committee on Cancer
TNM: Tumor-node-metastasis
OS: Overall survival
DSS: Disease-specific survival
aHR: Adjusted hazard ratio
CI: Confidence interval

## References

[1] Siegel RL, Miller KD, Jemal A (2019). Cancer statistics, 2019. CA Cancer J Clin.

[2] Rogers SN, Brown JS, Woolgar JA, Lowe D, Magennis P, Shaw RJ, Sutton D, Errington D, Vaughan D (2009). Survival following primary surgery for oral cancer. Oral Oncol.

[3] Woolgar JA, Rogers S, West CR, Errington RD, Brown JS, Vaughan ED (1999). Survival and patterns of recurrence in 200 oral cancer patients treated by radical surgery and neck dissection. Oral Oncol.

[4] Pfister DG, Spencer S, Adelstein D, Adkins D, Anzai Y, Brizel DM, Bruce JY, Busse PM, Caudell JJ, Cmelak AJ (2020). Head and neck cancers, version 2.2020, NCCN clinical practice guidelines in oncology. J Natl Compr Cancer Netw.

[5] Ivaldi E, Di Mario D, Paderno A, Piazza C, Bossi P, Iacovelli NA, Incandela F, Locati L, Fallai C, Orlandi E (2019). Postoperative radiotherapy (PORT) for early oral cavity cancer (pT1-2, N0-1): a review. Crit Rev Oncol Hematol.

[6] Lee CC, Su YC, Hung SK, Chen PC, Huang CI, Huang WL, Lin YW, Yang CC (2017). Recommendation for incorporation of a different lymph node scoring system in future AJCC N category for oral cancer. Sci Rep.

[7] Ebrahimi A, Clark JR, Zhang WJ, Elliott MS, Gao K, Milross CG, Shannon KF (2011). Lymph node ratio as an independent prognostic factor in oral squamous cell carcinoma. Head Neck.

[8] Lemieux A, Kedarisetty S, Raju S, Orosco R, Coffey C (2016). Lymph node yield as a predictor of survival in pathologically node negative oral cavity carcinoma. Otolaryngol Head Neck Surg.

[9] Divi V, Chen MM, Nussenbaum B, Rhoads KF, Sirjani DB, Holsinger FC, Shah JL, Hara W (2016). Lymph node count from neck dissection predicts mortality in head and neck cancer. J Clin Oncol.

[10] Gil Z, Carlson DL, Boyle JO, Kraus DH, Shah JP, Shaha AR, Singh B, Wong RJ, Patel SG (2009). Lymph node density is a significant predictor of outcome in patients with oral cancer. Cancer.

[11] Chen MM, Harris JP, Hara W, Sirjani D, Divi V (2016). Association of postoperative radiotherapy with survival in patients With N1 oral cavity and oropharyngeal squamous cell carcinoma. JAMA Otolaryngol Head Neck Surg.

[12] D'Cruz AK, Vaish R, Kapre N, Dandekar M, Gupta S, Hawaldar R, Agarwal JP, Pantvaidya G, Chaukar D, Deshmukh A (2015). Elective versus therapeutic neck dissection in node-negative oral cancer. N Engl J Med.

[13] Chang TS, Chang CM, Ho HC, Su YC, Chen LF, Chou P, Lee CC (2013). Impact of young age on the prognosis for oral cancer: a population-based study in Taiwan. PLoS ONE.

[14] Lin CY, Fan KH, Lee LY, Hsueh C, Yang LY, Ng SH, Wang HM, Hsieh CH, Lin CH, Tsao CK (2020). Precision adjuvant therapy based on detailed pathologic risk factors for resected oral cavity squamous cell carcinoma: long-term outcome comparison of CGMH and NCCN guidelines. Int J Radiat Oncol Biol Phys.

[15] Rajappa SK, Maheshwari U, Ram D, Koyyala VPB, Mandal G, Goyal S, Kumar R, Dewan AK (2019). Early oral cavity cancer: the prognostic factors and impact of adjuvant radiation on survival. Head Neck.

[16] Shrime MG, Gullane PJ, Dawson L, Kim J, Gilbert RW, Irish JC, Brown DH, Goldstein DP (2010). The impact of adjuvant radiotherapy on survival in T1-2N1 squamous cell carcinoma of the oral cavity. Arch Otolaryngol Head Neck Surg.

[17] Kao J, Lavaf A, Teng MS, Huang D, Genden EM (2008). Adjuvant radiotherapy and survival for patients with node-positive head and neck cancer: an analysis by primary site and nodal stage. Int J Radiat Oncol Biol Phys.

[18] Iocca O, Di Maio P, De Virgilio A, Pellini R, Golusinski P, Petruzzi G, Zocchi J, Pirola F, Janczak R, Golusinski W (2020). Lymph node yield and lymph node ratio in oral cavity and oropharyngeal carcinoma: preliminary results from a prospective, multicenter, international cohort. Oral Oncol.

[19] Mallen-St Clair J (2017). Quality metrics in oral cavity cancer-developing standards for optimal lymph node yield. JAMA Otolaryngol Head Neck Surg.

[20] Calabrese L, Giugliano G, Bruschini R, Ansarin M, Navach V, Grosso E, Gibelli B, Ostuni A, Chiesa F (2009). Compartmental surgery in tongue tumours: description of a new surgical technique. Acta Otorhinolaryngol Ital.

[21] Calabrese L, Bruschini R, Giugliano G, Ostuni A, Maffini F, Massaro MA, Santoro L, Navach V, Preda L, Alterio D (2011). Compartmental tongue surgery: long term oncologic results in the treatment of tongue cancer. Oral Oncol.

[22] Piazza C, Grammatica A, Montalto N, Paderno A, Del Bon F, Nicolai P (2019). Compartmental surgery for oral tongue and floor of the mouth cancer: oncologic outcomes. Head Neck.

