# Supplementary material for: Postoperative radiotherapy is associated with improved survival in pT1-2N1 oral and oropharyngeal cancer without adequate neck dissection
Source: Radiat Oncol. 2021 Jan 6;16:6. doi: 10.1186/s13014-020-01736-8 (PMC7788971; doi:10.1186/s13014-020-01736-8)
Supplement: Supplementary file 1 — Additional file 1: The relationship and multivariate analyses regarding 5-year overall and disease-free survivals between different quality of neck dissection. [file 13014_2020_1736_MOESM1_ESM.docx]

| Supplementary table 1. The relationship between postoperative radiotherapy and observation groups in 5-year overall and disease-free survivals among different quality of neck dissection. | | | | | | | | | | | | | | |
| --- | --- | --- | --- | --- | --- | --- | --- | --- | --- | --- | --- | --- | --- | --- |
|  | LNs<18 | | | |  | 18≦LNs<25 | | | |  | 25≦LNs | | | |
|  | Total | Event (%) | Survival rate (%) | *P* value |  | Total | Event (%) | Survival rate (%) | *P* value |  | Total | Event (%) | Survival rate (%) | *P* value |
| **Oral cavity (n=1,108)** |  |  |  |  |  |  |  |  |  |  |  |  |  |  |
| **Overall Survival** | 360 | 168 (46.7) | 48.9 | 0.287 |  | 185 | 69 (37.3) | 57.7 | 0.369 |  | 563 | 214 (38.0) | 55.2 | 0.403 |
| Without RT | 168 | 83 (49.4) | 46.6 |  |  | 73 | 30 (41.1) | 53.5 |  |  | 217 | 78 (35.9) | 58.3 |  |
| With RT | 192 | 85 (44.3) | 50.9 |  |  | 112 | 39 (34.8) | 60.4 |  |  | 346 | 136 (39.3) | 53.1 |  |
| **Disease-Specific Survival** | 360 | 89 (24.7) | 69.4 | 0.426 |  | 185 | 42 (22.7) | 73.1 | 0.960 |  | 563 | 137 (24.3) | 69.3 | 0.064 |
| Without RT | 168 | 44 (26.2) | 68.4 |  |  | 73 | 16 (21.9) | 72.8 |  |  | 217 | 43 (19.8) | 75.0 |  |
| With RT | 192 | 45 (23.4) | 70.2 |  |  | 112 | 26 (23.2) | 73.2 |  |  | 346 | 94 (27.2) | 65.7 |  |
|  |  |  |  |  |  |  |  |  |  |  |  |  |  |  |
| **Oropharynx (n=657)** |  |  |  |  |  |  |  |  |  |  |  |  |  |  |
| **Overall Survival** | 238 | 47 (19.7) | 78.4 | <0.001 |  | 104 | 19 (18.3) | 79.1 | 0.014 |  | 315 | 49 (15.6) | 80.9 | 0.134 |
| Without RT | 76 | 28 (36.8) | 58.7 |  |  | 32 | 10 (31.3) | 63.0 |  |  | 132 | 25 (18.9) | 77.0 |  |
| With RT | 162 | 19 (11.7) | 87.3 |  |  | 72 | 9 (12.5) | 85.6 |  |  | 183 | 24 (13.1) | 83.5 |  |
| **Disease-Specific Survival** | 238 | 20 (8.4) | 90.1 | 0.001 |  | 104 | 5 (4.8) | 94.8 | 0.534 |  | 315 | 27 (8.6) | 89.6 | 0.235 |
| Without RT | 76 | 12 (15.8) | 79.2 |  |  | 32 | 2 (6.3) | 93.2 |  |  | 132 | 14 (10.6) | 86.8 |  |
| With RT | 162 | 8 (4.9) | 94.5 |  |  | 72 | 3 (4.2) | 95.5 |  |  | 183 | 13 (7.1) | 91.5 |  |
| Abbreviation: LNs, lymph nodes; RT, radiotherapy. | | | | | | | | | | | | | | |

| Supplementary table 2. Multivariate analyses of risk factors regarding 5-year overall and disease-free survivals using Cox regression model among oral cavity patients different quality of neck dissection , *n*=1,108. | | | | | | | | |
| --- | --- | --- | --- | --- | --- | --- | --- | --- |
|  | LNs<18 | |  | 18≦LNs<25 | |  | 25≦LNs | |
|  | HR (95% C.I) | *P* value |  | HR (95% C.I) | *P* value |  | HR (95% C.I) | *P* value |
| **Overall Survival** |  |  |  |  |  |  |  |  |
| With RT | 0.81 (0.60–1.10) | 0.181 |  | 0.93 (0.57–1.51) | 0.763 |  | 1.04 (0.78–1.39) | 0.783 |
| Male | 0.90 (0.67–1.23) | 0.512 |  | 0.93 (0.57–1.53) | 0.783 |  | 1.20 (0.90–1.59) | 0.216 |
| Age | 1.03 (1.02–1.05) | <0.001 |  | 1.04 (1.02–1.06) | 0.001 |  | 1.01 (1.00–1.02) | 0.058 |
| Pathological T classification：T2 | 1.67 (1.23–2.27) | 0.001 |  | 1.76 (1.07–2.90) | 0.026 |  | 1.76 (1.32–2.33) | <0.001 |
| Differentiation：Poorly | 1.16 (0.81–1.68) | 0.419 |  | 1.76 (1.03–3.02) | 0.038 |  | 1.35 (0.99–1.83) | 0.057 |
|  |  |  |  |  |  |  |  |  |
| **Disease-Specific Survival** |  |  |  |  |  |  |  |  |
| With RT | 0.81 (0.53–1.24) | 0.330 |  | 1.17 (0.62–2.21) | 0.630 |  | 1.27 (0.88–1.84) | 0.202 |
| Male | 0.77 (0.51–1.18) | 0.229 |  | 1.09 (0.57–2.08) | 0.792 |  | 1.13 (0.79–1.60) | 0.509 |
| Age | 1.02 (1.00–1.04) | 0.027 |  | 1.03 (1.00–1.06) | 0.030 |  | 1.00 (0.99–1.01) | 0.969 |
| Pathological T classification：T2 | 1.68 (1.10–2.56) | 0.015 |  | 1.98 (1.04–3.79) | 0.039 |  | 1.61 (1.13–2.29) | 0.009 |
| Differentiation：Poorly | 1.21 (0.74–1.99) | 0.441 |  | 1.93 (0.98–3.80) | 0.059 |  | 1.41 (0.96–2.06) | 0.078 |
| *Adjusted for age, gender, pathological T classification, differentiation, site, race, marital and insurance.  **Abbreviation: LNs, lymph nodes; RT, radiotherapy. | | | | | | | | |

| Supplementary table 3. Multivariate analyses of risk factors regarding 5-year overall and disease-free survivals using Cox regression model among Oropharynx patients different quality of neck dissection , *n*=657. | | | | | | | | |
| --- | --- | --- | --- | --- | --- | --- | --- | --- |
|  | LNs<18 | |  | 18≦LNs<25 | |  | 25≦LNs | |
|  | HR (95% C.I) | *P* value |  | HR (95% C.I) | *P* value |  | HR (95% C.I) | *P* value |
| **Overall Survival** |  |  |  |  |  |  |  |  |
| With RT | 0.25 (0.14–0.46) | <0.001 |  | 0.25 (0.10–0.66) | 0.005 |  | 0.68 (0.39–1.21) | 0.189 |
| Male | 0.70 (0.38–1.29) | 0.250 |  | 1.98 (0.45–8.70) | 0.367 |  | 0.62 (0.34–1.15) | 0.129 |
| Age | 1.04 (1.01–1.06) | 0.009 |  | 1.05 (1.003–1.10) | 0.036 |  | 1.03 (0.99–1.05) | 0.066 |
| Pathological T classification：T2 | 1.35 (0.75–2.44) | 0.313 |  | 3.70 (1.20–11.45) | 0.023 |  | 1.55 (0.87–2.76) | 0.134 |
| Differentiation：Poorly | 1.16 (0.65–2.10) | 0.614 |  | 0.42 (0.15–1.15) | 0.090 |  | 0.77 (0.43–1.36) | 0.367 |
|  |  |  |  |  |  |  |  |  |
| **Disease-Specific Survival** |  |  |  |  |  |  |  |  |
| With RT | 0.24 (0.10–0.58) | 0.002 |  | 0.36 (0.06–2.26) | 0.276 |  | 0.64 (0.30–1.38) | 0.254 |
| Male | 0.60 (0.24–1.49) | 0.270 |  | 0.62 (0.05–7.12) | 0.699 |  | 0.56 (0.25–1.26) | 0.163 |
| Age | 1.00 (0.96–1.05) | 0.937 |  | 1.05 (0.96–1.16) | 0.276 |  | 0.99 (0.95–1.03) | 0.565 |
| Pathological T classification：T2 | 0.97 (0.39–2.40) | 0.942 |  | 119,290,000 (0.00–.) | 0.996 |  | 1.14 (0.53–2.46) | 0.734 |
| Differentiation：Poorly | 1.77 (0.71–4.39) | 0.218 |  | 0.59 (0.09–3.72) | 0.570 |  | 1.22 (0.55–2.70) | 0.626 |
| *Adjusted for age, gender, pathological T classification, differentiation, site, race, marital and insurance.  **Abbreviation: LNs, lymph nodes; RT, radiotherapy. | | | | | | | | |
